# Multi-marker analysis of circulating tumor cells in localized intermediate/high-risk and metastatic prostate cancer

**DOI:** 10.1007/s10585-024-10313-2

**Published:** 2024-09-21

**Authors:** Eva Welsch, Lilli Bonstingl, Barbara Holzer, Eva Schuster, Esther Weiß, Alexandru-Teodor Zaharie, Michael Krainer, Michael B. Fischer, Amin El-Heliebi, Robert Zeillinger, Eva Obermayr

**Affiliations:** 1 Molecular Oncology Group, Department of Obstetrics and Gynecology, Comprehensive Cancer Center, Medical University of Vienna, Waehringer Guertel 18-20, Vienna, 1090 Austria; 2 Division of Cell Biology, Histology and Embryology, Gottfried Schatz Research Centre, Medical University of Graz, Graz, Austria; 3 European Liquid Biopsy Society (ELBS), Hamburg, Germany; 4 OncoLab Diagnostics GmbH, Wiener Neustadt, Austria; 5 Department of Radiation Oncology, Medical University of Vienna, Vienna, Austria; 6 Division of Oncology, Department for Medicine I, Medical University of Vienna, Vienna, Austria; 7 Department of Blood Group Serology and Transfusion Medicine, Medical University of Vienna, Vienna, Austria; 8 Department for Biomedical Research, Center of Experimental Medicine, Danube University Krems, Krems an der Donau, Austria; 9 Biotechmed, Graz, Austria

**Keywords:** Circulating tumor cells, Prostate cancer, Liquid biopsy, qPCR, Gene expression, Parsortix

## Abstract

Circulating tumor cells (CTCs) are an established prognostic marker in metastatic prostate cancer (PrC) but have received little attention in localized high-risk disease. Peripheral blood was obtained from patients with early intermediate and high-risk PrC (*n* = 15) at baseline, after radiotherapy, and during follow-up, as well as from metastatic PrC patients (*n* = 23). CTCs were enriched using the microfluidic Parsortix^®^ technology. CTC-related marker were quantified with qPCR and RNA in-situ hybridization (ISH). Positivity and associations to clinical parameters were assessed using McNemar test, Fisher Exact test or log-rank test. The overall positivity was high in both cohorts (87.0% metastatic vs. 66.7% early at baseline). A high concordance of qPCR and RNA ISH was achieved. In metastatic PrC, *PSA* and *PSMA* were prognostic for shorter overall survival. In early PrC patients, an increase of positive transcripts per blood sample was observed from before to after radiation therapy, while a decrease of positive markers was observed during follow-up. CTC analysis using the investigated qPCR marker panel serves as tool for achieving high detection rates of PrC patient samples even in localized disease. RNA ISH offers the advantage of confirming these markers at the single cell level. Employing the clinically relevant marker PSMA, our CTC approach can be used for diagnostic purposes to screen patients profiting from PSMA-directed PET-CT or PSMA-targeted therapy.

**Supplementary Information:**

The online version contains supplementary material available at 10.1007/s10585-024-10313-2.

## Introduction

Prostate cancer (PrC), the most frequent cancer and third cause of cancer death in men in Europe, accounted for 22.3% of all new cancer cases and 10.0% of all cancer-related deaths in 2020 [1]. The 5-year relative survival rate of localized or regional disease at presentation is more than 99%; however with distant metastases just about 34% of the patients will survive five years [2].

The development of distant metastases is facilitated by the dissemination of tumor cells into the blood stream, known as circulating tumor cells (CTC). CTCs have prognostic relevance in different tumor types including PrC. The CellSearch^®^ assay (Menarini Silicon Biosystems) is currently the only US Food and Drug Administration (FDA) approved system for the detection of CTCs in metastatic PrC. This assay relies on the presence of epithelial characteristics such as the expression of EpCAM on the cell surface and intracellular cytokeratins. Using the CellSearch^®^ assay, an unfavorable pretreatment count of at least five CTCs per 7.5 ml blood was observed in 57% of patients with metastatic (castration-resistant) PrC and predicted shorter overall survival [3]. At early PrC stages CTCs were observed in just 8–33% of the cases, and the CTC numbers were lower than in patients with metastatic disease, ranging from 1 to 15 CTCs (per 7.5 ml blood) depending on the method used [4–7]. Although the presence of a single CTC detected by the CellSearch^®^ assay was shown to be linked to shorter overall survival of the patients with localized PrC [8], the clinical value of CTCs in this patient group has remained less conclusive than in patients with metastatic disease.

The detection of such rare numbers of CTCs depends on highly sensitive methods. In order to thrive the clinical applicability of CTC analysis in early PrC, significant progress is necessary on the technological side. One approach is the combination of different cell based assays (the CellSearch^®^, the CellCollector^®^ (GILUPI), and the EPISPOT), which have been shown to more than double the CTC positivity rate, reaching a cumulative positivity of 79% [9].

Molecular assays for CTC analysis based on gene expression of epithelial, neuroendocrine and PrC specific marker represent an interesting alternative to conventional immunofluorescent (IF) based assays and may lead to a higher sensitivity. In addition, qPCR allows the analysis of multiple genes of interest in a single sample. A multiplex gene expression analysis has achieved a higher positivity rate compared to cell-based assays in non-metastatic PrC patients and furthermore revealed a substantial heterogeneity in the captured CTCs from each patient [10]. Recently, a tailored PrC gene panel allowed the stratification of metastatic PrC patients’ survival and therapy response [11]. The hereby used label-free Parsortix^®^ (Angle plc.) CTC isolation technology, based on size and deformability was approved by the FDA for the capture and harvest of CTCs in metastatic breast cancer [12] and offers great potential also for PrC CTC analysis.

In previous studies we established a workflow for the detection and molecular characterization of CTCs in ovarian, breast, and lung cancer employing Parsortix^®^ and qPCR for the detection of CTC-related transcripts [13–16]. In the present study we investigated our approach in blood samples taken from patients with metastatic and localized PrC and investigated a panel of transcripts potentially related to the epithelial (*EpCAM*,* CK19*) and neuroendocrine (*DLL3*,* CHGA*,* SYP*) cell lineage, or transcripts specific for prostate (cancer) cells (*PSA*,* PSMA*,* AR*,* ERG*,* ERCC1*,* AMACR*,* ETV1*, and *KLK2*). The primary aim of our study was to test the feasibility of label-free (EpCAM independent) CTC enrichment and qPCR-based detection of potentially clinically relevant targets, as well as CTC detection using IF staining in patients with localized disease, at baseline, after radiotherapy and during follow-up until up to one year after therapy completion. A secondary aim was to evaluate our approach in a metastatic PrC cohort and confirm the assay by RNA hybridization (ISH).

## Materials and methods

### Patients and blood samples

The study included patients with metastatic prostate cancer (PrC) (*n* = 23) recruited at the Division of Oncology, Department of Medicine I, Vienna General Hospital, Austria and patients with primary localized PrC with unfavorable intermediate or high-risk (*n* = 15) recruited at the Department of Radiology, Vienna General Hospital, Austria (patient characteristics see Supplementary Table S1 and S2). Patients with unfavorable intermediate or high risk received 3D guided volumetric ac (VMAT) definitive radiotherapy with 60 Gy to the prostate and 46 Gy to the pelvic lymph nodes delivered in 20 fractions. Hormonal treatment was administered based on risk stratification for 6 months up to two years. The risk-stratification was done according to the D’Ámico risk stratification [17] and also using the corresponding ISUP groups, especially for the Gleason Score 7 (4 + 3) [18]. Additionally, 10 healthy male volunteers without any history of cancer were recruited at the Department of Blood Group Serology and Transfusion Medicine, Medical University of Vienna, Austria as healthy blood donors (HD). All patients and donors gave their written informed consent for the analysis of their specimen. This study was approved by the Ethics Committee of the Medical University of Vienna, Austria (EK366/2003, EK1966-2020, and EK2266/2018).

### Blood collection

Two different kind of blood collection tubes were used, Vacuette^®^ K_3_EDTA tubes (Greiner Bio-One, Austria) and CellSave™ Preservative tubes (Menarini Silicon Biosystems, US). The first milliliters of blood drawn after venipuncture were discarded in order to avoid contaminating skin cells in the blood samples. From all patients and HD, peripheral blood was drawn aseptically in three 9 ml Vacuette^®^ K_3_EDTA tubes for CTC enrichment with Parsortix^®^ and subsequent qPCR analysis. In addition, blood was taken in 9 ml CellSave™ Preservative tubes for IF evaluation of the Circulating Epithelial cell nPAC™ RUO Kit (Axon Dx, US). From the primary PrC patients, paired blood samples were taken before radiotherapy, at completion and every three months during follow-up for up to one year. From the metastatic PrC patients, the blood was taken at the time of progression between palliative treatment lines.

### Microfluidic CTC isolation and RNA isolation

For subsequent molecular analyses, the blood samples collected in Vacuette^®^ K_3_EDTA tubes were processed within four hours using the Parsortix^®^ device (Angle plc., UK) employing the microfluidic GEN3D6.5 cell separation cassette at 99mbar pressure. After the separation was completed, the captured cells were harvested. Immediately after cell harvest, the cells were partly transferred to poly-l-lysine glass slides and partly lysed by adding RLT lysis buffer (Qiagen, Germany). The lysates were stored at -80 °C until RNA extraction with the RNeasy Micro Kit (Qiagen, Germany) without DNase treatment according to manufacturer’s instructions. The total RNA was eluted in 14 µl RNase free water.

### Preamplification and qPCR using hydrolysis probes

Half of the total RNA volume was reverse transcribed using the SuperScript VILO Mastermix (Invitrogen, USA) (25 °C for 10 min, 42 °C for 1 h, 85 °C for 5 min, 4 °C). A pre-amplification was carried out on the 2720 Thermal Cycler (Applied Biosystems, USA) (25 °C for 5 min, 95 °C for 10 min, 10 cycles with 95 °C for 15 s and 60 °C for 4 min). The targets of interest (*EpCAM*,* CK19*,* PSA*,* PSMA*,* AR*,* CHGA*,* SYP*,* DLL3*,* ERG*,* ERCC1*,* AMACR*,* ETV1*, and *KLK2*), as well as the reference gene *CDKN1B* were quantified using the TaqMan Universal Mastermix II (ThermoFisher, USA) and exon spanning TaqMan assays (ThermoFisher, USA) in duplicates after the target-specific pre-amplification step on the QuantStudio™ 7 Flex Real-Time PCR System (ThermoFisher, USA) with standard thermal cycling conditions (50 °C for 2 min, 95 °C for 10 min, 40 cycles of 95 °C for 15 s and 60 °C for 1 min).

### RT-qPCR using hybridization probes

The remaining half of the total RNA volume was used to determine *CK19* gene expression using FRET probes and primers hybridizing to the *CK19* gene [19]. The one step RT-qPCR was carried out with the PrimeScript III Mastermix (Takara) at the LightCycler 480 II (Roche) (52 °C for 5 min, 95 °C for 10 s, followed by 50 cycles of 95 °C for 5 s and 60 °C for 30 s and one cooling cycle with 40 °C for 30 s).

### Cut-off values for qPCR analysis

Cut-off threshold values were calculated for transcripts that were also detected in HD samples, in order to define positive patient samples. For each transcript, the threshold was calculated by subtracting an integer multiple of the standard deviation from the mean Ct-value of the HD samples. A patient sample with a Ct-value below the calculated threshold value was defined as positive for the respective transcript. If a transcript was not detected in any HD sample, any amplification in patient samples showing a Ct-value ≤ 35 (Cq-value ≤ 45 for CK19 FRET probe assay) was defined as positive.

### RNA *in-situ* hybridization

The poly-l-lysine slide with spotted cells after Parsortix^®^ separation was used to visualize mRNA as described previously [20, 21] with in-situ padlock probe hybridization. In short, cDNA was generated using target specific reverse transcription primers. Padlock probes were then hybridized to the cDNA. After ligation, circularized padlock probes were amplified by rolling circle amplification and visualized by hybridizing fluorescently labelled detection probes. Divergent from previous publications, targets were labelled with two-color combinations to increase specificity. All genes were targeted by multiple reverse transcription primers and padlock probes for increased sensitivity, and additional transcripts were visualized: hematopoietic markers ((*PTPRC* (*CD45*), *ITGAM* (*CD11B*), *FCGR3A* and *FCGR3B* (*CD16*), *CD4*, *ITGB2* (*CD18*)), epithelial (*EpCAM*,* KRT8*,* KRT18*,* KRT19*) and prostate specific tumor markers (*PSA*, *PSMA*, *AR-FL*, *AR-V7*), neuroendocrine-associated markers (*SYP*,* CHGA*,* NCAM1*,* DLL3*,* SLFN11*), and *VIM*. Cells without hematopoietic markers and at least one in-situ signal for epithelial, prostate specific, or neuroendocrine markers were identified as potential CTCs. The evaluated slides were scanned in 40x magnification. A detailed method description is given in [22].

### Immunofluorescent staining of CTCs

6 ml peripheral blood drawn in a CellSave**™** tube was used for CTC enrichment and staining with the Circulating Epithelial cell nPAC™ RUO Kit (Axon Dx, US) employing anti-panCK, anti-CK19, anti-CD45 and other antibodies directed against proprietary white blood cell markers (WBC) and DAPI (4′,6-diamidino-2-phenylindole) for nuclear staining. The procedure was performed according to the manufacturer’s instruction. The stained cells were scanned (40x magnification) with the nCyte Dx^®^ platform (Axon Dx, LLC). Image acquisition and evaluation was performed with the AI-based nCyte Dx nAble^®^ software.

### Statistics

Fisher’s exact test was used to evaluate the association between the marker positivity and patients’ characteristics. Survival outcomes were compared with Kaplan-Meier survival analyses and log-rank testing. The period of time in months between blood draw and either death or the last date the patient was seen alive was defined as overall survival (OS). McNemar test was used to compare the individual transcripts and CTC positivity of early PrC patients before and after therapy. Two-sided tests were used at all analyses. Statistical analysis was performed with IBM SPSS Statistics 21. Immunofluorescent images were evaluated and represented with Inkscape 1.2. Graphics design was carried out with GraphPad (version 10.1.0) and R (version: 4.3.2).

## Results

### CTCs in primary localized PrC before and after radiotherapy

Fifteen primary PrC patients were recruited to investigate CTCs in serial blood samples taken before (baseline) and after radiation therapy. A total of 58 samples were drawn from the primary PrC cohort. Ten from these patients received hormone therapy in addition to radiation therapy. The baseline characteristics of the patients are given in the Supplementary Table S1.

**Fig. 1:**
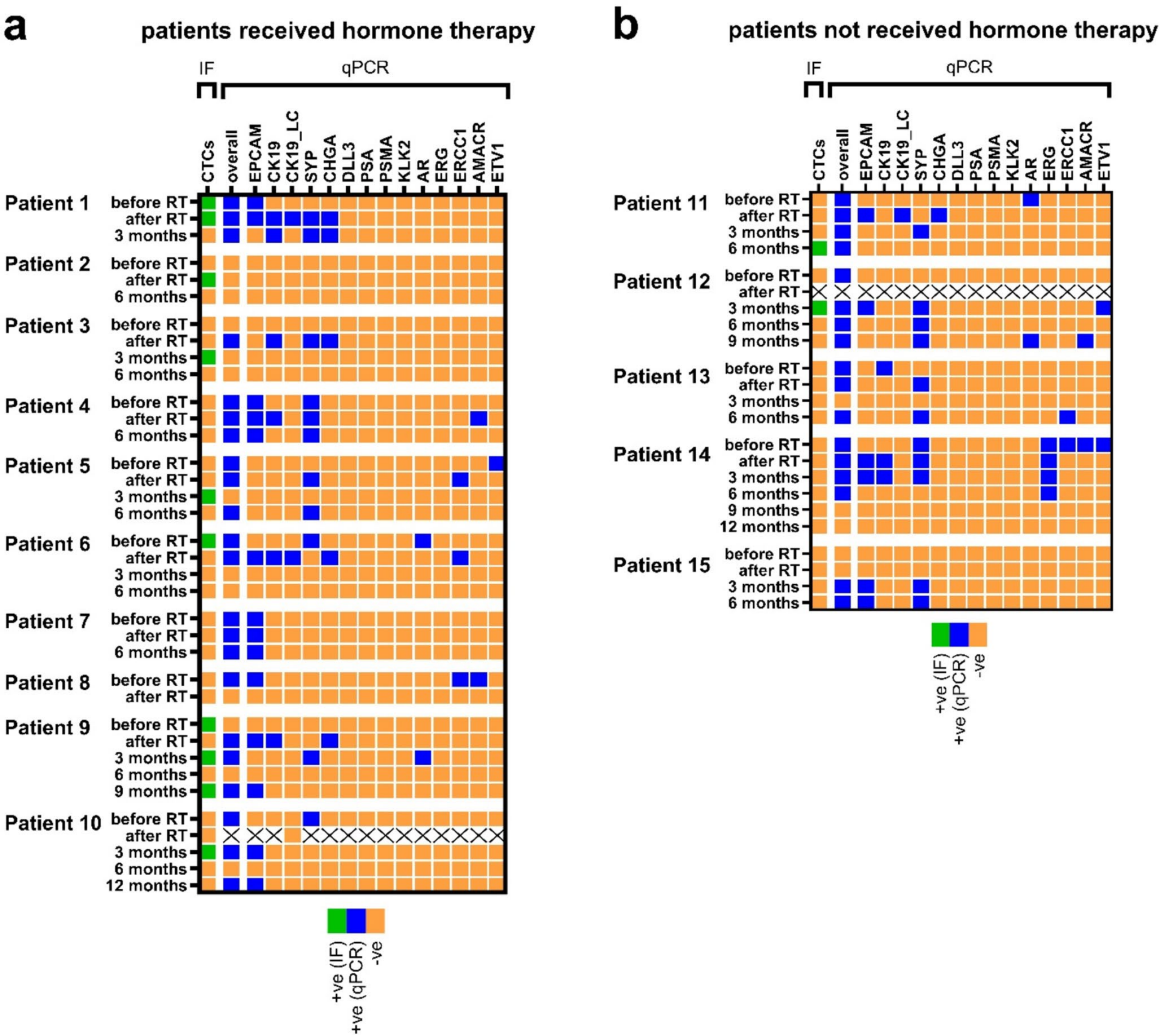
Heatmap depicting positive and negative early PrC patients’ samples at time points before and after radiation therapy (RT), as well as after 3, 6, 9 or 12 months after treatment completion determined by qPCR CTC marker or CTC immunofluorescent (IF) staining. Patients that additionally received hormone therapy are shown at the left side (**a**) and patients that only received radiation therapy are shown on the right side (**b**) of the graph. qPCR positive samples (blue) were defined after applying a cut-off threshold. The detection of minimum one CTC was defined as detection limit for IF positive (green) samples. Negative samples by qPCR or IF are depicted in orange. Samples not available are marked with a cross (hydrolysis probe qPCR of patient 10 sample after radiotherapy could not be evaluated due to low expression of reference gene)

A panel of epithelial (*EpCAM*,* CK19)*, neuroendocrine *(CHGA*,* SYP*,* DLL3)* and PrC- specific (*PSA*,* PSMA*,* AR*,* ERG*,* ERCC1*,* AMACR*,* ETV1*, and *KLK2*) CTC-related transcripts were investigated by qPCR. We observed a high positivity of transcripts in blood samples taken before and after radiotherapy. Considering a threshold of one transcript, the overall positivity after treatment was 76.9% and 66.7% at baseline (McNemar; *p* = 1.000). In addition, one out of 10 HD samples was positive for the transcripts *ERCC1* and *SYP*. Using a more stringent threshold of three markers, the positivity was 14.3% before and 50.0% after therapy (Mc Nemar; *p* = 0.125). In this case none of the HD samples was positive. Positivity of the highly specific transcripts not detected in HD (e.g. *CK19*_LC and *CHGA*) were observed after radiation therapy but not at baseline (Supplementary Table S2). In the blood samples taken at serial time points during follow-up, the number of positive transcripts declined again (Fig. 1). The results of the two *CK19* based assays were concordant in 86.2% of all investigated blood samples (Prevalence-adjusted bias-adjusted kappa PABAK = 0.724). As the assay is highly specific (no expression in HD), *CK19*_LC positive samples (5.0%) will most likely contain epithelial CTCs [23]; nevertheless, *CK19* TaqMan™ qPCR achieved a higher positivity rate (15.5%) due to the target-specific pre-amplification step.

**Fig. 2:**
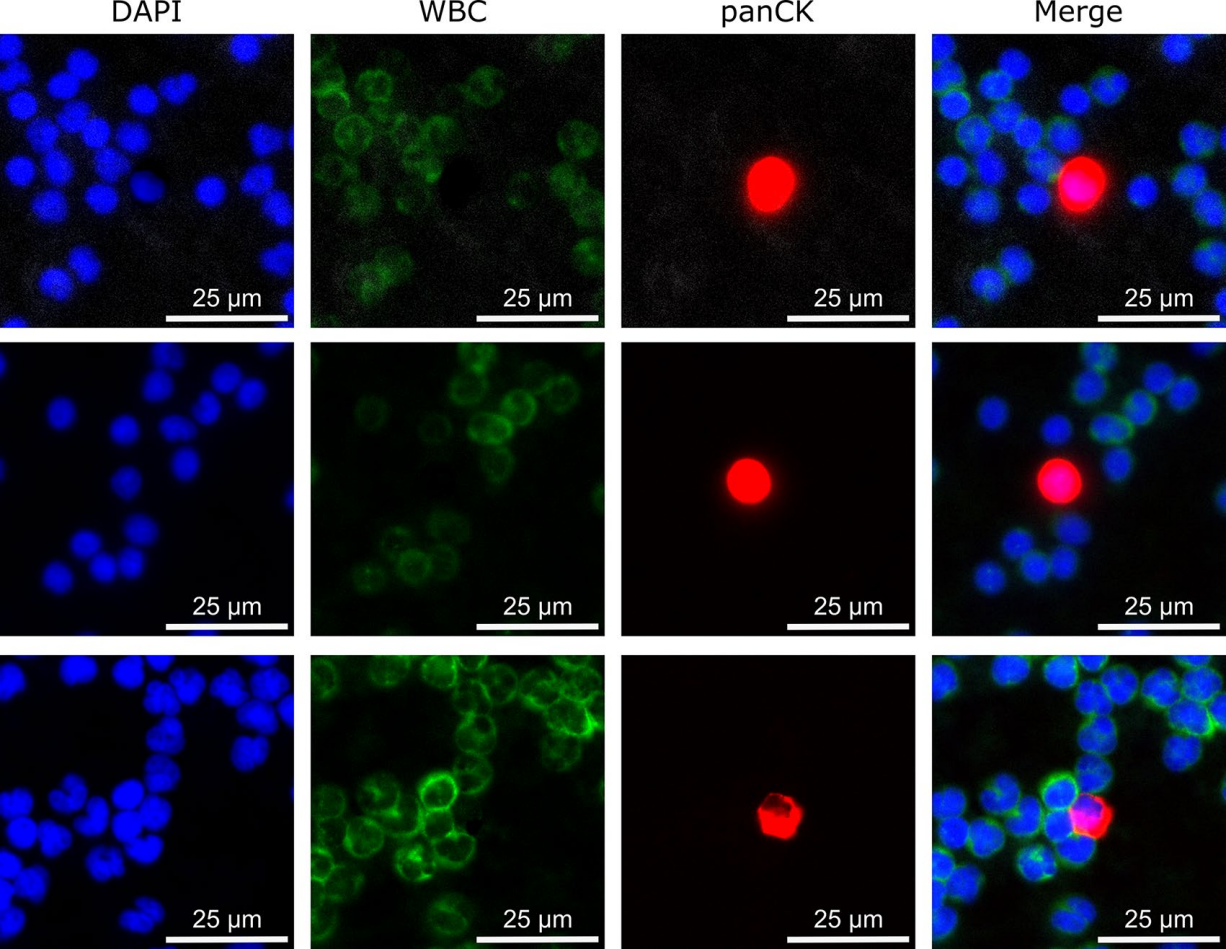
Example images of three different CTCs detected in early PrC patients by immunofluorescent staining of panCK (red), white blood cell (WBC) (green) and nuclear counterstaining using DAPI; A panCK positive, DAPI positive and WBC negative cell was defined as CTC; The given scale bar in each individual image marks the size of 25 μm

In all samples from early PrC patients, low CTC counts were detected by IF staining, ranging from 1 to 58 cells. CTCs were found in just 20.0% (3/15) of the samples taken before and 14.3% (2/14) of the samples collected after radiotherapy. No statistical difference between the positivity rates at the two time points was observed (McNemar *p* = 1.000). In addition, CTCs were detected in six follow-up samples. Example images of IF stained cells are shown in Fig. 2.

Neither the analyzed markers nor the presence of epithelial CTCs detected by IF was associated with Gleason score or outcome of the patients at study end (see Supplementary Table S1). Within the observation period (range 17–28 months from baseline), no disease progression occurred in any patient; therefore Kaplan-Meier analysis was not performed. Nevertheless, as shown in the heatmap (Fig. 1), CTC detection by IF staining and qPCR could have additive value in terms of CTC positivity.

### CTC detection by qPCR and clinical evaluation in metastatic PrC

The same CTC-related markers were investigated in microfluidic enriched blood samples from 23 patients with metastatic PrC. The cohort comprised patients with advanced prostate cancer, all of whom experienced recurrence following local therapy, which included either radical prostatectomy or definitive radiotherapy. All patients were recurrences and the majority of patients were castration-resistant at the time of analysis. Among the cohort, six patients presented with biochemical recurrence only, and one patient had local recurrence without distant spread. Nine patients exhibited bone metastases exclusively, while two patients had isolated lymph node metastases. Additionally, five patients had both bone and lymph node involvement. Notably, none of the patients in this cohort had developed visceral metastases. Detailed patient characteristics are given in Supplementary Table S3.

Overall, 87.0% of the patients were scored positive for at least one marker. Similar to the blood sample cohort from early PrC patients, the results of the two *CK19* based assays were concordant in 78.3% of the cases (PABAK = 0.565). The PrC specific transcripts *PSA*, *PSMA* and *KLK2*, as well as *DLL3*, were exclusively detected in blood samples from patients with metastatic but not with localized disease (Supplementary Table S2).

**Fig. 3:**
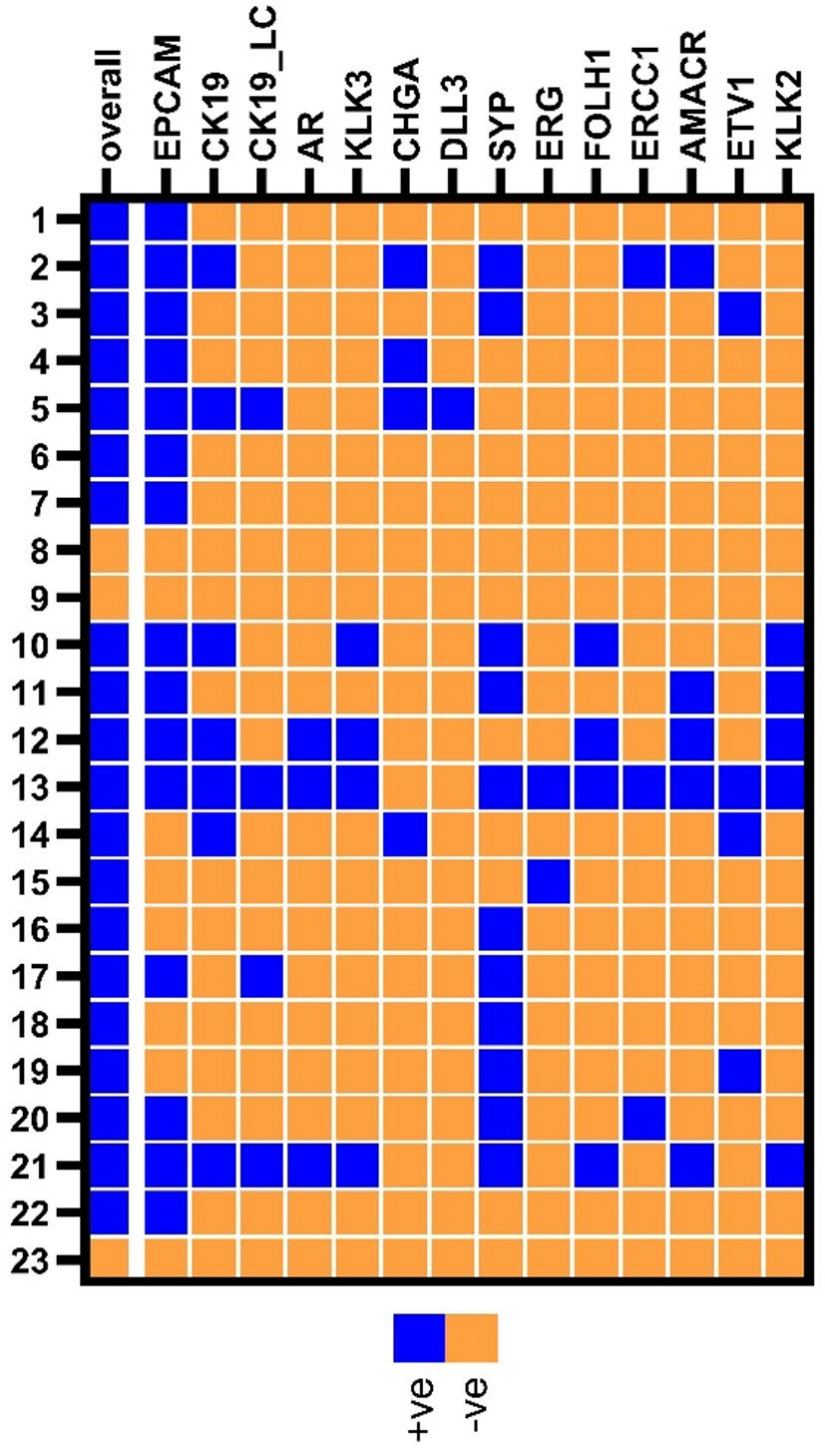
Heatmap depicting positive (blue) and negative (orange) metastatic PrC patient (*n* = 23) for qPCR CTC marker. qPCR positive samples were defined after applying a cut-off threshold

**Fig. 4:**
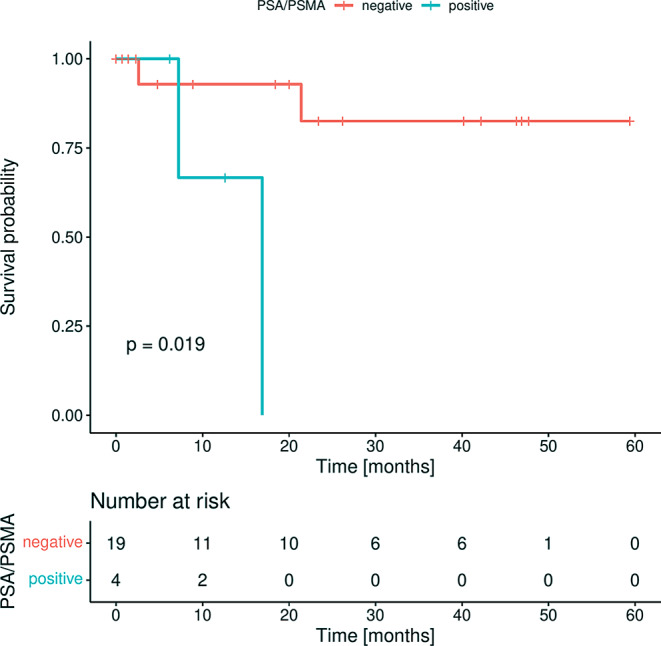
Kaplan-Meier plot for overall survival (OS) of metastatic prostate cancer patients according to *PSA* and *PSMA* positivity (blue) and negativity (red) in CTCs; Log-rank testing was used for comparing the patient’s outcome; *p* < 0.05 is defined as level of significance

By investigating the association of the analyzed markers to clinical parameters, we observed that *PSA* and *PSMA* were positive in the same patients and that the positivity of these markers (Fisher’s exact test; both *p* = 0.014) and of *AR* (Fisher’s exact test; *p* = 0.047) was significantly related to the presence of metastasis in the bone (Supplementary Table S3). Within the observation period (median 20 months from blood draw), four patients died (Fig. 3, patients 8, 10, 20, 21). The concordant positivity of *PSA* and *PSMA* (17.4%) was associated to shorter overall survival of metastatic PrC patients (log rank test; *p* = 0.020, Fig. 4).

### Comparison of CTC detection methods in metastatic PrC

In five of the initially recruited 23 metastatic PrC patients (Fig. 3, patients 1–5), the presence of CTC-related transcripts were additionally analyzed by ISH and CTCs were visualized by IF staining.

**Fig. 5:**
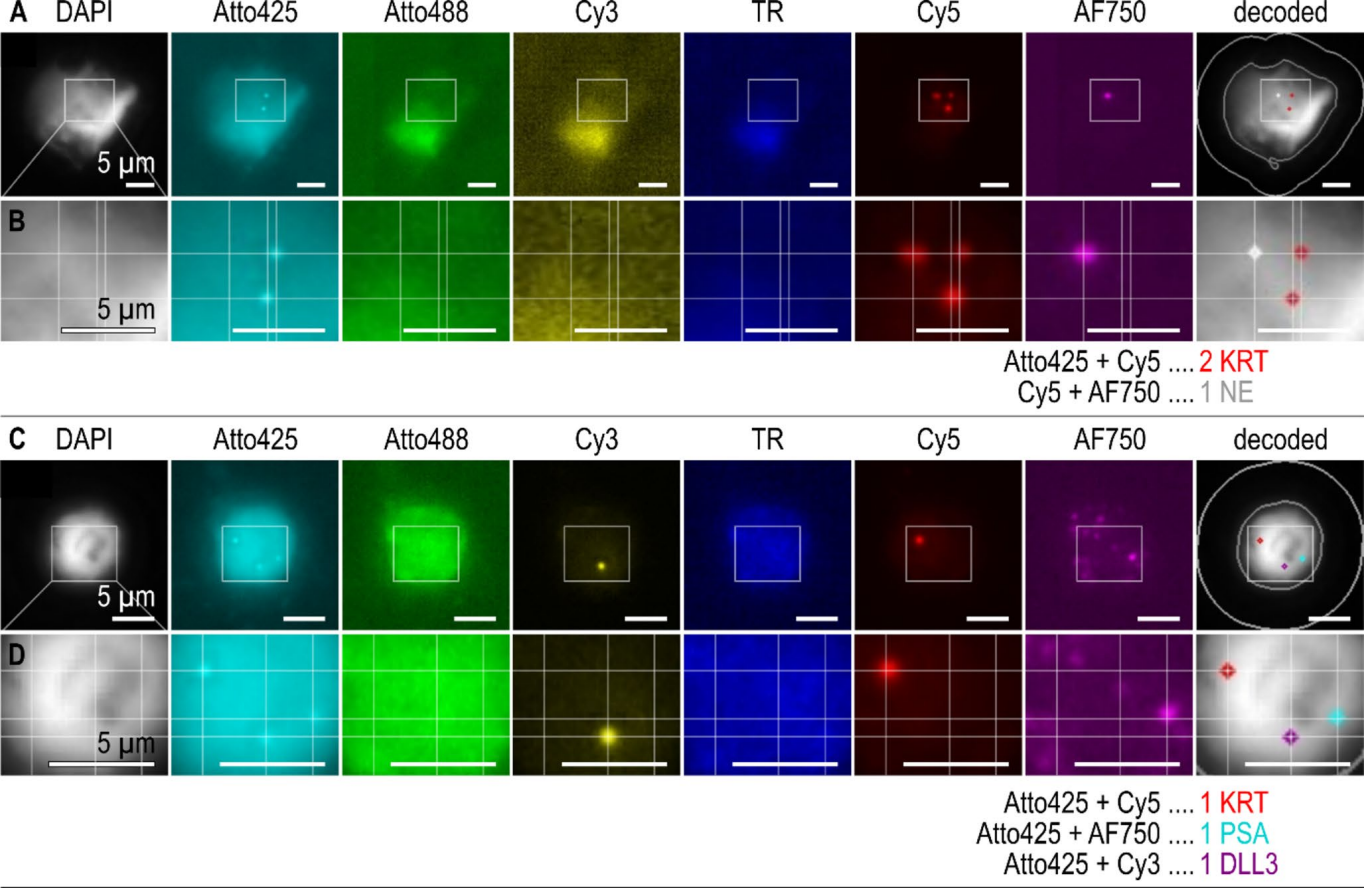
Example images of two CTCs of metastatic PrC patient 2 detected by dual-color in-situ hybridization with (**A**-**B**) coexpression of cytokeratin (*KRT*) and neuroendocrine markers (NE (pool of *CHGA*,* SYP*,* NCAM1*)), and (**C**-**D**) coexpression of cytokeratin (*KRT*), prostate specific antigen (*PSA*) and neuroendocrine marker *DLL3*. In-situ signals were decoded based on their color code (e.g. colocalized Atto425 and Cy5 signals are decoded as *KRT*). Pseudocolored images are depicted for each channel, followed by a DAPI image with decoded in-situ signals and outlines of nucleus and cell border. Areas marked by white rectangles (**A**, **C)** are shown with higher magnification (**B**, **D**), grid lines for orientation help to identify and decode colocalized in-situ signals. The given scale bar in each individual image marks the size of 5 μm

Using ISH we were able to prove the presence of epithelial, neuroendocrine and PrC- specific transcripts at a single cell level (Fig. 5). A 100% concordance of qPCR and ISH was achieved by evaluating neuroendocrine transcripts *SYP*, *CHGA* or *DLL3*. For the epithelial transcripts *CK19* and/or *EpCAM* a concordance of 80% was obtained. CTC IF staining resulted in a lower positivity than RNA based methods (see Supplementary Fig. S1).

## Discussion

Enumeration and characterization of CTCs offer a valuable tool for disease monitoring of PrC. To implement the analysis of CTCs into the clinical routine, still a significant progress has to be made on the technological side detecting low CTC numbers occurring in early stages [4–6]. Compared to conventional IF staining, qPCR offers higher CTC detection rates by investigating a broad spectrum of CTC-related transcripts including epithelial, neuroendocrine and prostate specific markers. Using our established qPCR marker panel for CTC detection after microfluidic enrichment, we were able to achieve a 66.7% positivity of early PrC patient samples at baseline. After completion of radiotherapy an increase of positive CTC markers – especially the epithelial (*CK19*, *EpCAM*) and neuroendocrine (*SYP*, *CHGA*) transcripts was observed, which led us to the hypothesis of increased CTC shedding due to the disruption of the tumor by radiation therapy. Tumor disruption by radiotherapy was previously proven by elevated levels of radiation-induced DNA double strand break markers in CTCs [24]. During follow-up a decrease of positive markers indicated response to therapy. Longer follow-up data will enable clinical analysis of longitudinal data and reveal more information based on the CTC change during therapy.

The established qPCR transcript panel was also evaluated in a cohort of metastatic PrC patients. We were able to detect CTC related transcripts in 87.0% of peripheral blood samples. In a subset of five metastatic PrC patient samples, we confirmed the qPCR results with RNA ISH by visualizing the same transcripts on an individual cell level. Thereby, we achieved a high concordance of qPCR and RNA ISH regarding the positivity of epithelial and neuroendocrine transcripts. In addition, the presence of CTCs was assessed by IF staining. The lower CTC positivity rates obtained by IF staining than by qPCR can be explained by the difference in RNA- and protein-based detection methods. Both methods provide valuable information about patients’ CTC status; nevertheless, qPCR may have additive value because multiple clinically relevant markers can be assessed simultaneously. An inclusion of RNA- and protein-based mesenchymal specific markers could further characterize the phenotype of these CTCs at the molecular level in further studies.

Despite the advantages of the highly sensitive qPCR-based CTC detection approach, it comes with some drawbacks. For example, it is not possible to depict CTC morphological differences or cell cluster, which can provide additional information on the patient’s disease status. Also, other cancer-associated cell types are not detected. In such cases, RNA- and protein-based IF staining methods provide benefits over qPCR-based methods. Nevertheless, it proves useful in predicting patients’ survival and detecting therapy-relevant transcripts.

In the present study, the PrC-specific markers *PSA*, *PSMA*, and *KLK2* were only detected in blood samples from patients with metastatic disease, but not with early localized PrC. Although *KLK2* positivity was not linked to any clinical parameter in our study, it has been previously associated with poor survival [25]. *KLK2* has already been shown to have clinical utility and is used in the 4Kscore^®^ Test to predict the risk of PrC aggressiveness [26]. *KLK3* encodes the PSA protein, which is commonly used as a serum marker in PrC. In the present study, the two PrC specific marker *PSA* and *PSMA* were associated to shorter overall survival. *PSA* is an important diagnostic marker with high specificity used in PrC monitoring [27]. The theranostically relevant marker PSMA (prostate-specific membrane antigen, often referred to as *FOLH1*) has been shown to be significantly higher expressed in primary PrC than in benign tissue, and even higher in distant metastases and metastatic lymph nodes compared to primary tumors [28]. CTC analysis of PSMA protein using IF was previously shown to be associated with poor OS in metastatic PrC patients [29]. PSMA has diagnostic potential, as it is used for the highly sensitive and specific targeted PET-CT. FDA approved PSMA-targeting drugs, such as the radioligand agent Lutetium-177 PSMA-617 (trade name: Pluvicto) offer personalized treatment of PrC patients. The drug delivers a small amount of radioactive Lutetium-177 attached to a molecule that specifically binds to PSMA overexpressing PrC cells, thereby killing the cells [30–32]. As these diagnostic and therapeutic procedures are cost-intensive, a pre-screening of patients profiting from PSMA-PET-CT and PSMA targeted therapy is necessary. CTC-related analysis might be a promising and comparably cheap tool for monitoring of PrC patients, in order to define patient groups for targeted diagnosis and therapy.

## Conclusion

Our study points to the power of the cost-effective qPCR analysis of various PrC type specific, epithelial and neuroendocrine transcripts in liquid biopsies in order to detect CTCs at a high sensitivity and to further allow the molecular characterization thereof. RNA ISH offers the advantage of confirming qPCR results at a single cell level. CTC analysis is a promising diagnostic tool for early PrC patient cohorts. Disease recurrences might be detected earlier by longitudinal monitoring of easily obtained liquid biopsies. The detection of the prognostic prostate specific transcripts *PSA* and *PSMA* can be evaluated in CTCs to stratify patients profiting from targeted diagnosis and therapy.

## Electronic supplementary material

Below is the link to the electronic supplementary material.

Supplementary Material 1

## Data Availability

The data underlying this article will be shared on reasonable request to the corresponding author.
