# Supplementary material for: Multi-marker analysis of circulating tumor cells in localized intermediate/high-risk and metastatic prostate cancer
Source: Clin Exp Metastasis. 2024 Sep 21;41(6):937–45. doi: 10.1007/s10585-024-10313-2 (PMC11607046; doi:10.1007/s10585-024-10313-2)
Supplement: Supplementary file 1 — Supplementary Material 1 [file 10585_2024_10313_MOESM1_ESM.docx]

**Supplementary Information**

**Multi-marker Analysis of Circulating Tumor Cells in Localized Intermediate/High-Risk and Metastatic Prostate Cancer**

**Clinical and Experimental Metastasis**

Eva Welsch ^1^, Lilli Bonstingl ^2,3^, Barbara Holzer ^1^, Eva Schuster ^1^, Esther Weiß ^4^, Alexandru-Teodor Zaharie ^5^, Michael Krainer ^6^, Michael B. Fischer ^7,8^, Amin El-Heliebi ^2,3,9^, Robert Zeillinger ^1,4^, and Eva Obermayr ^1^

**Affiliations**

^1^Molecular Oncology Group, Department of Obstetrics and Gynecology, Comprehensive Cancer Center, Medical University of Vienna, Vienna, Austria; EO: [0000-0001-6324-2961](https://orcid.org/0000-0001-6324-2961), EW: [0009-0008-3560-1769](https://orcid.org/0009-0008-3560-1769), RZ: [0000-0001-6771-4591](https://orcid.org/0000-0001-6771-4591)

^2^Division of Cell Biology, Histology and Embryology, Gottfried Schatz Research Centre, Medical University of Graz, Graz, Austria; AE: [0000-0002-7679-6856](https://orcid.org/0000-0002-7679-6856); LB: [0009-0009-0757-6962](https://orcid.org/0009-0009-0757-6962)

^3^European Liquid Biopsy Society (ELBS), Hamburg, Germany

^4^OncoLab Diagnostics GmbH, Wiener Neustadt, Austria

^5^Department of Radiation Oncology, Medical University of Vienna, Vienna, Austria; ATZ: [0000-0002-8697-9058](https://orcid.org/0000-0002-8697-9058)

^6^Division of Oncology, Department for Medicine I, Medical University of Vienna, Vienna, Austria; MK: [0000-0002-7011-4957](https://orcid.org/0000-0002-7011-4957)

^7^Department of Blood Group Serology and Transfusion Medicine, Medical University of Vienna, Austria; MBF: [0000-0002-5876-0243](https://orcid.org/0000-0002-5876-0243)

^8^Department for Biomedical Research, Center of Experimental Medicine, Danube University Krems, Krems an der Donau, Austria

^9^Biotechmed, Graz, Austria

**Corresponding author**

Eva Obermayr

Medical University of Vienna, Waehringer Guertel 18-20, 1090 Vienna, Austria

eva.obermayr@muv.ac.at

T: +43 (1)40400 78270; F: +43 (1)40400 78320

**Table of contents:**

Table S1

Table S2

Table S3

Figure S1

References

# Results

Table S1: Characteristics of the 15 localized prostate cancer patients and positivity rates of qPCR CTC marker and immunofluorescently (IF) stained CTC before the start of radiotherapy. The associations of marker positivity and patients’ characteristics were evaluated using a Fisher’s exact test.

|  |  | qPCR | | | | | | | | | | | | | | | IF |
| --- | --- | --- | --- | --- | --- | --- | --- | --- | --- | --- | --- | --- | --- | --- | --- | --- | --- |
|  | n | overall | EPCAM | CK19 | CK19_LC | AR | PSA | CHGA | DLL3 | SYP | ERG | PSMA | ERCC1 | AMACR | ETV1 | KLK2 | CTCs |
| All Patients | 15 |  |  |  |  |  |  |  |  |  |  |  |  |  |  |  |  |
| Age (years)  Median  Range | 75.5  55.1 – 83.0 | -  - | -  - | -  - | -  - | -  - | -  - | -  - | -  - | -  - | -  - | -  - | -  - | -  - | -  - | -  - | -  - |
| GleasonScore  7  8  9  10 | 5  3  6  1 | 60.0%  66.7%  66.7%  100.0% | 0.0%  33.3%  33.3%  100.0% | 20.0%  0.0%  0.0%  0.0% | 0.0%  0.0%  0.0%  0.0% | 20.0%  0.0%  16.7%  0.0% | 0.0%  0.0%  0.0%  0.0% | 0.0%  0.0%  0.0%  0.0% | 0.0%  0.0%  0.0%  0.0% | 0.0%  33.3%  33.3%  100.0% | 0.0%  33.3%  0.0%  0.0% | 0.0%  0.0%  0.0%  0.0% | 0.0%  66.7%  0.0%  0.0% | 0.0%  66.7%  0.0%  0.0% | 20.0%  33.3%  0.0%  0.0% | 0.0%  0.0%  0.0%  0.0% | 20.0%  0.0%  33.3%  0.0% |
| *p* |  | 1.000 | 0.201 | 0.600 | - | 1.000 | - | - | - | 0.201 | 0.267 | - | 0.057 | 0.057 | 0.543 | - | 0.802 |
| Outcome  Alive  Dead | 13  2 | 76.9%  100.0% | 23.1%  50.0% | 7.7%  0.0% | 0.0%  0.0% | 7.7%  50.0% | 0.0%  0.0% | 0.0%  0.0% | 0.0%  0.0% | 23.1%  50.0% | 7.7%  0.0% | 0.0%  0.0% | 7.7%  50.0% | 7.7%  50.0% | 15.4%  0.0% | 0.0%  0.0% | 15.4%  50.0% |
| *p* |  | 1.000 | 1.000 | 1.000 | - | 0.257 | - | - | - | 0.476 | 1.000 | - | 0.257 | 0.257 | 1.000 | - | 0.371 |

Table S2: Overall positivity and individual positivity of CTC mRNA qPCR marker and immunofluorescently (IF) stained CTC of metastatic and early prostate cancer patients at the begin and end of therapy. qPCR marker positive samples were defined after applying a cut-off threshold. The detection of minimum one CTC (CK+, CD45- and DAPI+) was defined as detection limit for positive samples.

|  | metastatic | early (begin) | early (end) |
| --- | --- | --- | --- |
| qPCR_Overall | 20/23 (87.0%) | 10/15 (66.7%) | 10/13 (76.9%) |
| qPCR_EPCAM | 15/23 (62.5%) | 4/15 (26.4%) | 7/13 (53.8%) |
| qPCR_CK19 | 7/23 (30.4%) | 1/15 (6.7%) | 6/13 (46.2%) |
| qPCR_CK19_LC | 4//23 (17.4%) | 0/15 (0.0%) | 3/14 (21.4%) |
| qPCR_AR | 3/23 (13.0%) | 2/15 (13.3%) | 0/13 (0.0%) |
| qPCR_PSA | 4/23 (17.4%) | 0/15 (0.0%) | 0/13 (0.0%) |
| qPCR_CHGA | 4/23 (17.4%) | 0/15 (0.0%) | 5/13 (38.5%) |
| qPCR_DLL3 | 1/23 (4.3%) | 0/15 (0.0%) | 0/13 (0.0%) |
| qPCR_SYP | 11/23 (47.8%) | 4/15 (26.7%) | 6/13 (46.2%) |
| qPCR_ERG | 2/23 (8.7%) | 1/15 (6.7%) | 1/13 (7.7%) |
| qPCR_PSMA | 4/23 (17.4%) | 0/15 (0.0%) | 0/13 (0.0%) |
| qPCR_ERCC1 | 3/23 (13.0%) | 2/15 (13.3%) | 2/13 (15.4%) |
| qPCR_AMACR | 5/23 (21.7%) | 2/15 (13.3%) | 1/13 (7.7%) |
| qPCR_ETV1 | 4/23 (17.4%) | 2/15 (13.3%) | 0/13 (0.0%) |
| qPCR_KLK2 | 5/23 (21.7%) | 0/15 (0.0%) | 0/13 (0.0%) |
| IF_CTCs | 3/5 (60.0%) | 3/15 (20.0%) | 2/14 (14.3%) |

Table S3: Characteristics of the 23 metastatic prostate cancer patients and positivity rates of qPCR CTC marker. The associations of marker positivity and patients’ characteristics were evaluated using a Fisher’s exact test.

|  | n | overall | EPCAM | CK19 | CK19_LC | AR | PSA | CHGA | DLL3 | SYP | ERG | PSMA | ERCC1 | AMACR | ETV1 | KLK2 |
| --- | --- | --- | --- | --- | --- | --- | --- | --- | --- | --- | --- | --- | --- | --- | --- | --- |
| All Patients | 23 |  |  |  |  |  |  |  |  |  |  |  |  |  |  |  |
| Age (years)  Median  Range | 70.0  55.8 – 82.1 | -  - | -  - | -  - | -  - | -  - | -  - | -  - | -  - | -  - | -  - | -  - | -  - | -  - | -  - | -  - |
| Site of Metastasis  Bone only  Lymph only  Bone & Lymph  Occult  Local | 9  2  5  6  1 | 88.9%  50.0%  80.0%  100.0%  100.0% | 77.8%  50.0%  60.0%  66.7%  0.0% | 55.6%  0.0%  20.0%  16.7%  0.0% | 33.3%  0.0%  0.0%  16.7%  0.0% | 33.3%*  0.0%  0.0%  0.0%  0.0% | 44.4%*  0.0%  0.0%  0.0%  0.0% | 11.1%  50.0%  20.0%  16.7%  0.0% | 11.1%  0.0%  0.0%  0.0%  0.0% | 44.4%  0.0%  80.0%  33.3%  100.0% | 22.2%  0.0%  0.0%  0.0%  0.0% | 44.4%*  0.0%  0.0%  0.0%  0.0% | 22.2%  0.0%  20.0%  0.0%  0.0% | 33.3%  0.0%  40.0%  0.0%  0.0% | 11.1%  0.0%  20.0%  16.7%  100.0% | 44.4%  0.0%  20.0%  0.0%  0.0% |
| *p* |  | 0.487 | 0.640 | 0.430 | 0.619 | 0.385 | 0.170 | 0.802 | 1.000 | 0.266 | 0.609 | 0.170 | 0.726 | 0.446 | 0.384 | 0.311 |
| Castration Resistant  Yes  No | 19  4 | 84.2%  100.0% | 68.4%  50.0% | 36.8%  0.0% | 15.8%  25.0% | 15.8%  0.0% | 21.1%  0.0% | 15.8%  25.0% | 5.3%  0.0% | 42.1%  75.0% | 10.5%  0.0% | 21.1%  0.0% | 15.8%  0.0% | 26.3%  0.0% | 15.8%  25.0% | 26.3%  0.0% |
| *p* |  | 1.000 | 0.589 | 0.273 | 1.000 | 1.000 | 1.000 | 1.000 | 1.000 | 0.317 | 1.000 | 1.000 | 1.000 | 0.539 | 1.000 | 0.539 |
| Outcome  Alive  Dead | 19  4 | 89.5%  75.0% | 63.2%  75.0% | 26.3%  50.0% | 15.8%  25.0% | 10.5%  25.0% | 10.5%  50.0% | 21.1%  0.0% | 5.3%  0.0% | 42.1%  75.0% | 10.5%  0.0% | 10.5%  50.0% | 10.5%  25.0% | 21.1%  25.0% | 21.1%  0.0% | 15.8%  50.0% |
| *p* |  | 0.453 | 1.000 | 0.557 | 1.000 | 0.453 | 0.125 | 1.000 | 1.000 | 0.317 | 1.000 | 0.125 | 0.453 | 1.000 | 1.000 | 0.125 |

*bone metastasis only is associated to marker positivity, p<0.05


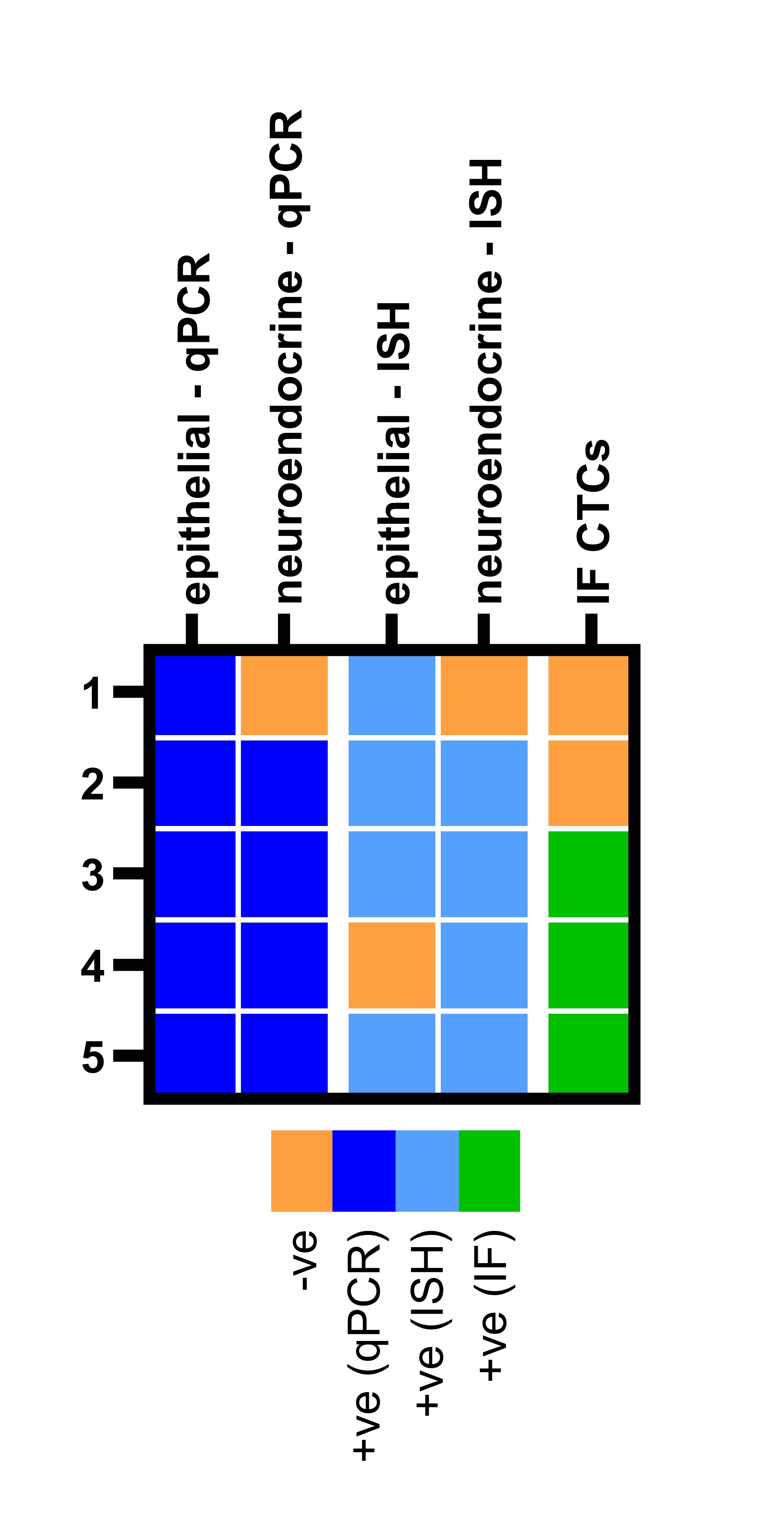


Figure S1: Heatmap depicting epithelial (CK19 and/or EpCAM) and neuroendocrine (SYP, CHGA, and/or DLL3) marker panel positivity of five metastatic PrC patients analyzed with qPCR and in-situ hybridization (ISH); as well as CTC detected by immunofluorescent staining (IF). qPCR positive samples after applying a cut-off threshold are depicted in dark blue, and ISH positive samples in light blue. A minimum of one CTC was defined as threshold for IF positivity (green). Negative samples are depicted in orange.

# References
